# An acute exercise at low to moderate intensity attenuated postprandial lipemia and insulin responses

**DOI:** 10.1016/j.jesf.2023.10.006

**Published:** 2023-10-31

**Authors:** Lisa L. Ji, Vicki S. Fretwell, Abel Escamilla, Wanxiang Yao, Tianou Zhang, Meizi He, John Q. Zhang

**Affiliations:** a Department of Kinesiology, The University of Texas at San Antonio, USA; b Department of Public Health, The University of Texas at San Antonio, USA

**Keywords:** Acute exercise, Triglyceride, Postprandial lipemia, Insulin resistance

## Abstract

**Objective:**

The purpose of this study was to investigate the effects of different exercise intensities on postprandial lipemia (PHTG) and insulin resistance in healthy individuals.

**Methods:**

Participants were 10 adult males with normal fasting triglyceride (TG) concentrations (age = 34 ± 2.8 y, body mass = 72.9 ± 2.4 kg, fasting plasma TG = 1.36 ± 0.18 mmol/l, VO_2max_ = 43.7 ± 3.0 ml/kg/min, fasting glucose = 5.2 ± 0.2 mmol/l and fasting Homeostatic Model Assessment for Insulin Resistance (HOMA2-IR) = 1.7 ± 0.3). In this study, each participant performed a control trial (Ctr, no exercise), and 3 exercise trials at 40 % (40%T), 60 % (60%T), and 70 % (70%T) of their VO_2max_. In the exercise trials, participant jogged on a treadmill for 1 h at a designated intensity. A fat-rich meal was consumed by each participant 12 h after exercise. Blood samples were taken at 0 h (before the meal), and 2 h, 4 h, 6 h, 8 h, and 24 h after the meal. The plasma TG, area score under TG concentration curve over an 8 h-period (TG tAUC) after the meal, and HOMA2-IR were analyzed.

**Results:**

Our results showed that at 2 h, 4 h, and 6 h after the meal, TG in all exercise trials were lower than Ctr (p < 0.05) but did not differ from each other. All the exercise trials were lower in TG tAUC scores than Ctr (p < 0.02), but differences were not observed among the exercise trials. In comparison to Ctr, a significant difference in HOMA2-IR in both 60 % T and 70 % T (p < 0.05 and p < 0.01, respectively) was observed, but not in 40 % T.

**Conclusion:**

The results suggest that exercising at low to moderate exercise intensity for 1 h sufficiently attenuates a fat meal induced PHTG. Moderate exercise intensity also effectively mitigates insulin resistance.

## 1 Introduction

Cardiovascular disease (CVD) is a leading cause of death worldwide. Postprandial hypertriglyceridemia (PHTG), a condition characterized by elevated levels of triglycerides after a meal, is a common lipid abnormality associated with an increased risk of CVD.^1^ PHTG is characterized with elevated TG-rich lipoproteins, which include partially catabolized chylomicrons containing ApoB-48 and/or increased hepatic production of very low-density lipoproteins (VLDL-TG) containing ApoB-100, after a meal.^2^ Prolonged high levels of TG-rich lipoproteins contribute to endothelial dysfunction and insult to the arterial wall. Therefore, exaggerated PHTG response indicates poor TG clearance from the blood stream and is often associated with atherosclerosis, insulin resistance, low-density lipoprotein (LDL), low levels of high-density lipoprotein cholesterol (HDL-C), and obesity.^3^^,^^4^ Individuals consuming high-fat diets may be exposed to PHTG for longer episodic periods.

PHTG may also induce thrombogenesis and stroke by causing elevated plasma viscosity, increased fibrinogen, and enhanced clotting.^5^ Individuals with hypertriglyceridemia (HTG) often have a prolonged PHTG after a fat meal and, therefore, are more susceptible to CVD. Insulin plays a major role of PHTG by promoting hepatic secretion of VLDL-TG. Insulin-resistant state is concomitantly associated with hyperinsulinemia and, therefore, results in elevated VLDL-TG production.^6^ Consequently, individuals with insulin resistance often demonstrate HTG and amplified prolonged PHTG.^7^ The engagement of exercise can elicit multiple benefits. Studies have shown that exercise training improves insulin sensitivity.^8^, ^9^, ^10^ Postprandial insulin resistance tends to decrease with exercise training^11^ but also with just a single bout of aerobic exercise^12^ and accumulated intermittent exercises.^13^^,^^14^ Similarly, PHTG was also attenuated with a single bout of aerobic exercise.^15^^,^^16^ In addition, multiple short bouts of exercise throughout the day also effectively reduced next day PHTG compared to one continuous exercise session.^17^^,^^18^ Not only were the effects observed on healthy individuals, but overweight participants were also able to display reductions in TG concentrations after exercise.^19^ After ingesting a fat-rich meal, healthy adolescent boys exhibited lower PHTG response when they participated in either continuous exercise or intermittent game activities.^20^ This poses the question of what exercise intensity elicits the most effective decrease in PHTG. Multiple studies have found different intensities to show a reduction in PHTG. Exercise intensity at 65 % VO_2max_ and intermittent walking were both found to be beneficial in reducing PHTG concentrations, while the former was more effective than the latter.^21^ High intensity interval sprint-type running at 100 % VO_2max_ was also seen to decrease PHTG concentrations.^22^^,^^23^ However, the concerns with implementing exercise at 100 % VO_2max_ is the dramatic increase rate in fatigue that may reduce the sustainability to thoroughly perform an exercise bout at 100 % VO_2max_.

Katsanos et al. reported that moderate-intensity exercise, performed 1 h before a fat meal, attenuates postprandial lipemia and this effect was not associated with postheparin lipoprotein lipase.^24^ Similarly, various studies^7^^,^^25^^,^^26^ demostrated the impact of an acute exercise bout on PHTG and reported positive effect on attenuation of postprandial-induced lipemia. However, these studies mainly employed a single exercise intensity. A rencent meta-analysis indicated that postprandial TG clearance was largely dependent on exercise energy expenditure.^27^ This finding suggests that different amount caloric expendure from different exercise intensites may affect PHTG response. Indeed, one of our previous findings revealed that exercising at 40–70 % intensities not only attenuated PHTG, but also lowered insulin response among physically inactive individuals with metabolic syndrome.^28^ However, the effects of different exercise intensities on PHTG and insulin resistance in healthy individuals (non-hyperlipidemic) remains to be elucidated. Thus, the objective of the study was to investigate the effects of various exercise intensities on PHTG and insulin resistance in healthy individuals.

## 2 Materials and methods

### 2.1 Participants and initial measurements

**Fig. 1:**
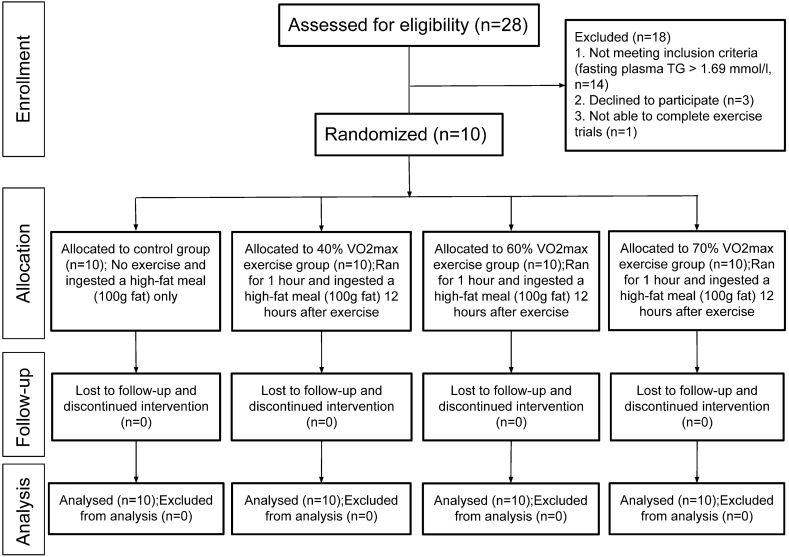
Consolidated Standards of Reporting Trials (CONSORT) diagram reporting enrollment, allocation, follow-up, and analysis of participants in the randomized controlled trial.

Ten healthy males aged 34 ± 2.8 years with normal blood lipid levels (fasting plasma TG ≤ 1.69 mmol/l) participated in the experiment (Table 1). The power calculation and sample size determination were based on the comparison of TG area scores in our previous study.^26^ The calculated effective sample size was 1.88. The sample size necessary to make this kind of study significant at p < 0.05 was about 10 subjects.^29^ The Consolidated Standards of Reporting Trials (CONSORT) diagram showing participants flow is shown in Fig. 1 and described in details as follows. The participants were informed of the risks associated with the study and were required to complete an informed consent form. The study protocol was approved by the University of Texas-San Antonio Institutional Review Board. Upon the initial visit, anthropometric measurements were obtained to provide baseline characteristics. Body mass index was calculated after measuring the participants’ height (m) and body mass (kg). Skin-fold caliper measurements taken at the chest, abdomen, and thigh were calculated to evaluate body fat percentage.^30^ Participants were of normal BMI, 23.3 ± 1 kg/m^2^ and possessed ideal body fat percentages, 13.6 ± 1.6 %. Blood samples were taken to measure fasting concentrations of triglycerides, total cholesterol, insulin, glucose, and insulin resistance (HOMA2-IR).^31^

**Table 1:** Participant characteristics (N = 10).

Participant characteristics	Means ± SE
Age, yrs	34 ± 2.8
Weight, kg	72.9 ± 2.4
Waist/Hip ratio	0.89 ± 0.01
Body mass index, kg/m^2^	23.3 ± 1.0
VO_2max,_ ml^.^ kg^−1^min^−1^	43.7 ± 3.0
Fasting TG, mmol/l	1.36 ± 0.18
Fasting TC, mmol/l	4.61 ± 0.34
Fasting HDL-C, mmol/l	1.07 ± 0.06
Fasting NEFA, mmol/l	0.21 ± 0.04
Fasting insulin, pmol/l	83.95 ± 10.05
Fasting glucose, mmol/l	5.2 ± 0.2
Fasting HOMA2-IR	1.7 ± 0.3

Values are means ± SE. TG, triglyceride; TC, total cholesterol; HDL-C, high-density lipoprotein cholesterol; NEFA, non-esterified fatty acids; HOMA2-IR, Homeostatic Model Assessment for Insulin Resistance.

### 2.2 Experimental design

Each of the qualified participants had a VO_2max_ test. Once the maximal threshold was established for each participant, they underwent 3 exercise trials at 40 %, 60 %, and 70 % of their VO_2max_. The participants ran on a treadmill at each respective exercise intensity for 1 h in duration. Fig. 1 illustrates the experimental flow chart. Participants acted as their own control with a trial consisting of no exercise (Ctr). The order of the trials was randomized to minimize potential testing bias. After each trial, participants were given 7–14 days to fully recover. Twelve hours after each exercise session, participants ingested a high-fat meal (100 g fat). Choosing a protocol that included exercise 12 h prior to fat loading was based on previous findings,^26^^,^^32^ which documented that exercising 12 h prior to fat loading more effectively attenuated postprandial lipemia than 24 h prior to or 1 h after a fat-meal intake. The exercise regiments were well tolerated by all participants.

**Fig. 2:**
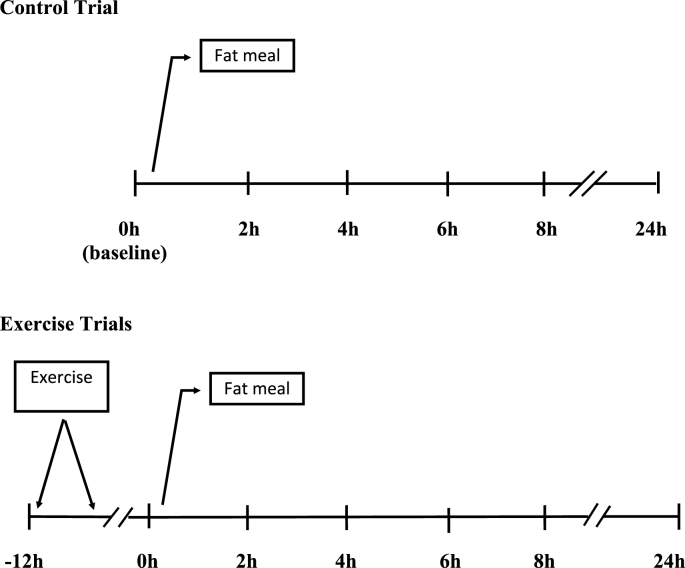
Trial flow chart. Control, fat-meal only; Three exercise trials were performed at 40 %, 60 %, or 70 % VO_2_max 12 h prior to a fat-meal intake. Trials were conducted 1–2 wks apart. Blood samples were drawn at 0 h, 2 h, 4 h, 6 h, 8 h, and 24 h.

Immediately before the fat meal ingestion (0 h), a baseline blood sample was collected. Blood samples were also obtained at 2 h, 4 h, 6 h, 8 h, and 24 h after the meal (Fig. 2). A standard normal meal (75 % carbohydrate, 16 % protein, and 9 % fat) consisting of a Subway sandwich (595 kcal, 76 g carbohydrate, 24 g protein, and 24 g fat), and one bag of Baked Lay's potato chips (120 calories, 26 g carbohydrate, 2 g protein, 5 g fat) was provided after the 8 h blood drawing. Plasma TG, the total area under the TG concentration curve scores (TG tAUC score), and insulin resistance (HOMA2-IR: homeostatic model assessment for insulin resistance; high score indicating insulin resistance) were analyzed. Participants also had a standard snack (five pieces of Honey Graham crackers containing 297 kcal, 52.5 g carbohydrate, 2.5 g protein, and 8.9 g fat) 12 h before the 24 h blood sample was taken. During the span of 24 h testing period, participants were only allowed to consume plain drinking water in combination with the provided standard food.

### 2.3 Preparatory dietary and physical activity control

To reduce intra-participant variability, each participant completed a 24 h dietary record during the day immediately prior to the first trial. Dietary intake data is shown in Table 2. A copy of this diet record was given back to the participant before each subsequent trial. To reduce dietary variation in lipemia, participants were required to replicate their diet during the 24 h period prior to each subsequent trial. Reminder calls were made to each participant two days before each trial instructing them to follow the same 24 h diet that they recorded prior to their first trial. Caffeine intake was not allowed 24 h prior to the trials, and exercise or alcohol intake was not allowed 3 days before the experimental trials. Telephone calls were made to each participant four days before each trial to remind him to restrain from exercising for three days prior to the trial. Time induced metabolic variations in consideration were kept minimal by administering the high-fat test at the same hour of the day. Exercising, other than the experimental trials, was not permitted during each 24 h testing period.

**Table 2:** Dietary intake prior to exercise trials.

Energy Components	Means ± SE	
Total Calories (Kcal)	2633.6 ± 170.3	
Carbohydrate (g)	350.4 ± 24.9	
Protein (g)	97.5 ± 3.8	
Fat (g)	93.6 ± 8.6	
CHO (%)	53.1 ± 0.6	
Protein (%)	15.3 ± 1.0	
Fat (%)	31.7 ± 1.1	

Values are means ± SE. Dietary energy intake before exercise trials (N = 10).

### 2.4 VO_2max_ test and exercise trials

A graded VO_2max_ treadmill test was administered to determine each participant's exercise intensities regarding percentage of VO_2max_. Briefly, participants warmed up for 5 min on a treadmill. After a 5-min warm-up on the treadmill, participants jogged at an initial speed of 4 mph for the first 2 min of the test. After the initial 2 min, the speed of the treadmill was increased every minute in increments of 0.5 mph until the treadmill speed was up to 6.5 mph. Thereafter, the speed remained constant, and the treadmill grade was raised by 2 % every minute until exhaustion.^26^ The following criteria were used to determine VO_2max_, oxygen uptake plateau, heart rate exceeding age-predicted maximal heart rate (220-age), or respiratory exchange ratio (RER) exceeding 1.10.

The required initial workloads to produce 40 %, 60 %, and 70 % of a participant's VO_2max_ were interpolated from the participant's VO_2max_ and workload obtained from the VO_2max_ test. Five minutes after starting exercise, the workload was adjusted accordingly to maintain the target level of oxygen uptake designated for an exercise trial throughout the exercise session. The average oxygen uptake and the respiratory exchange ratio of the steady state phase of each exercise session were used for caloric expenditure calculation.

### 2.5 Test meal

A participant was given a fat-rich meal in the form of a milkshake after 12 h fasting and consumed within 10 min in each trial. The milkshake consisted of a combination of 270 ml of whipping cream and 65 g of specialty ice cream with walnuts (980 kcal, 100 g fat, 17 g carbohydrate, and 3 g protein). This meal has been successfully used in previous studies to induce PHTG.^7^^,^^26^^,^^28^^,^^32^

### 2.6 Blood sampling and analysis

Ten milliliters of blood sample were collected by using a vacutainer containing EDTA. The blood samples were centrifuged at 2000g for 15 min at 4 °C for plasma separation. Plasmas were stored at −80 °C until analysis. Plasma TG and cholesterol concentrations were measured enzymatically using diagnostic kits (Infinity™ TG Reagent; Cholesterol reagent, procedure #353, Sigma, St. Louis, MO). Plasma glucose levels were measured using a diagnostic kit (Infinity™ Glucose Reagent, Sigma, St. Louis, MO, USA). Insulin levels were analyzed using a^125^I Radioimmunoassys (RIA) kit (ICN Pharmaceuticals, Costa Mess, CA, USA). The intra-assay coefficients of variation for TG, total cholesterol, HDL-C, glucose, and insulin were 1.5, 0.8, 1.4, 1.9, and 2.8 %, respectively. Non-esterified fatty acids (NEFA) were analyzed using a NEFA assay kit (Wako Diagnostics, Richmond, VA, USA). All plasma samples were diluted with 0.9 % saline (1:1) before precipitation. Insulin resistance was evaluated using the HOMA2-IR model,^31^ calculated with a HOMA2 calculator released by the Medical Sciences Division, University of Oxford: HOMA Calculator (https://www.rdm.ox.ac.uk/about/our-clinical-facilities-and-mrc-units/DTU/software/homa).

### 2.7 Data analysis

A two-way (trial x time) analysis of variance (ANOVA) with repeated-measures was performed to test the effects of exercise intensities on TG, insulin, and NEFA data. According to the trapezoidal rule, the magnitude of total TG response was also quantified as TG tAUC score.^26^ TG tAUC score is a conventional index indicating plasma TG response to a fat-meal intake. One-way ANOVA with repeated measure was performed to analyze data of TG tAUC score, fasting glucose, and HOMA2-IR. An ANOVA with significant F ratios (p < 0.05) was followed by Tukey post hoc tests. SigmaPlot software (version 11) was used to perform the analysis. All data (including figures) are reported as means ± SE.

## 3 Results

Table 1 displays the physical characteristics and the fasting lipidemic status of the participants. Table 3 shows the caloric expenditure and heart rate responses during the exercise sessions. Results not sharing the same English letter are significantly different from each other over the trials (p < 0.003). During the three trials, the caloric expenditures and mean heart rates were significantly different from each other. The percentage of carbohydrate expenditure at 40 % T, (78 ± 0.02 %), was significantly lower than that of both 60 % T, (88 ± 0.03 %), and 70 % T (94 ± 0.01 %). However, there was no significant difference in carbohydrate utilization between 60 % T and 70 % T. 40 % T depicted the highest fat utilization, 22 ± 0.02 %, among the three exercise trials (p < 0.003), whereas 60 % T and 70 % T did not differ in fat utilization.

**Table 3:** Metabolic heart rate responses during the exercise sessions.

Exercise Trials	Total Kcal/h	%Carbohydrate Kcal	%Fat Kcal	Average Heart Rate (b/min)
40%T	388 ± 29.1^a^	78 ± 0.02^a^	22 ± 0.02^a^	110 ± 2.7^a^
60%T	612 ± 45.1^b^	88 ± 0.03^b^	12 ± 0.03^b^	149 ± 3.9^b^
70%T	738 ± 57.5^c^	94 ± 0.01^b^	6 ± 0.01^b^	160 ± 3.8^c^

Values are means ± SE. Results not sharing the same English letter are significantly different from each other over the trials at p < 0.003.

**Fig. 3:**
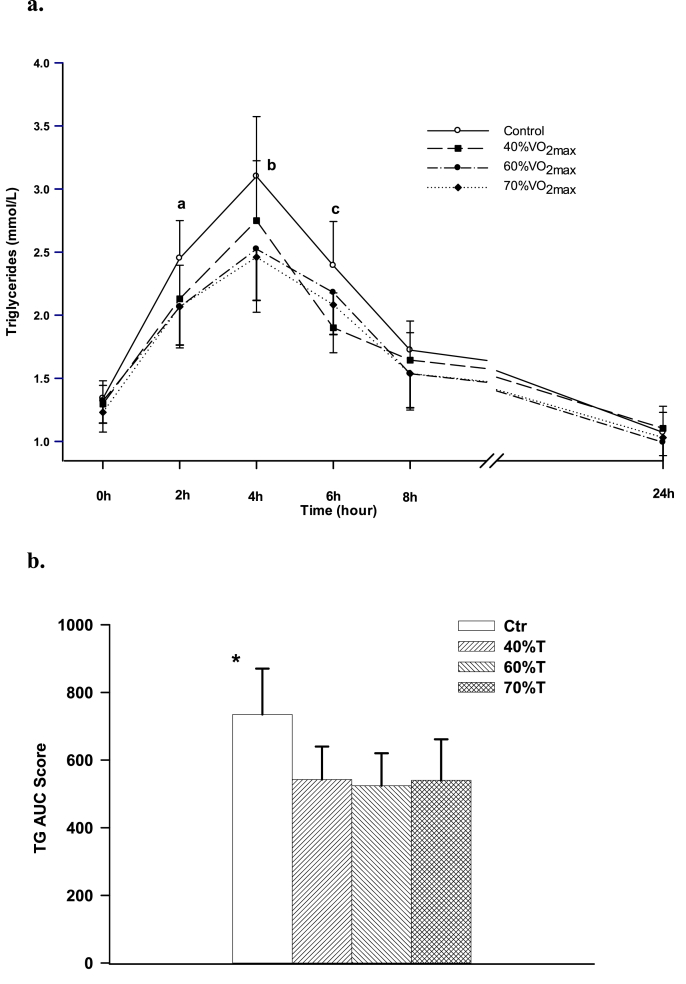
a: Effect of exercise intensities on plasma TG. Means not sharing a common English letter are significantly different from each other over the trials at P < 0.05. b: Effect of exercise intensities on plasma TG clearance. TG tAUC scores in the exerciser trials were lower than Ctr (*p < 0.02).

Fig. 3a displays the plasma TG concentration over time among the three exercise trials and Ctr. At 0 h (before the fat-meal intake), TG concentrations did not significantly differ among the four trials. At 2 h, 4 h, and 6 h after the meal, TG in all exercise trials were lower than Ctr (p < 0.05), but did not differ from each other at these time points. TG seemed to peak at 4 h after the meal in all of the trials. At the 6 h, and 8 h, and 24 h after the meal, the TG concentrations were similar for all trials. Fig. 3b illustrates the data of TG tAUC scores. All the exercise trials were lower in TG tAUC scores than Ctr (p < 0.02). However, there were no significant differences among the exercise trials. Nevertheless, the exercise trials had an average of 27 % TG tAUC score reduction compared to that of the control trial.

**Fig. 4:**
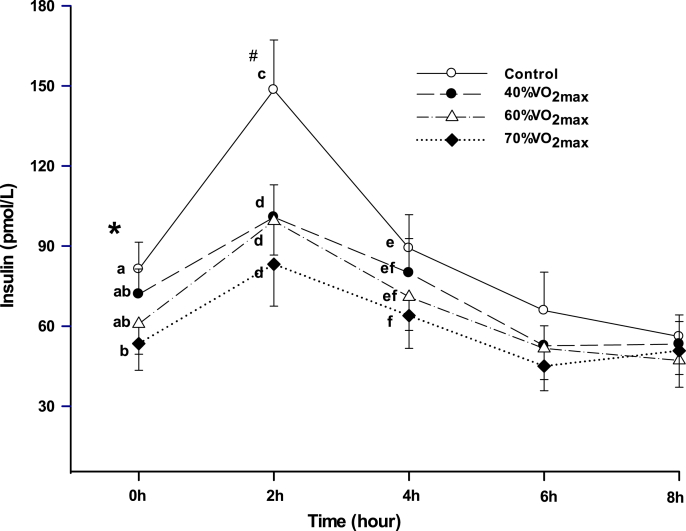
Effect of exercise intensities on insulin concentrations. Means not sharing a common English letter are significantly different from each other over the trials (p < 0.04). *p < 0.04; #p < 0.02.

Fig. 4 illustrates the effect of different exercise intensities on insulin concentrations of an 8 h period after a high fat meal ingestion. At 0 h, before the meal intake, only 70 % T had lower insulin concentration than Control (p < 0.04). At 2 h, all exercise trials were significantly lower than Control (p < 0.02). At 4 h, only 70 % T had lower insulin concentration than Control (p < 0.04). There were no significant differences in insulin concentrations among the exercise trials.

**Fig. 5:**
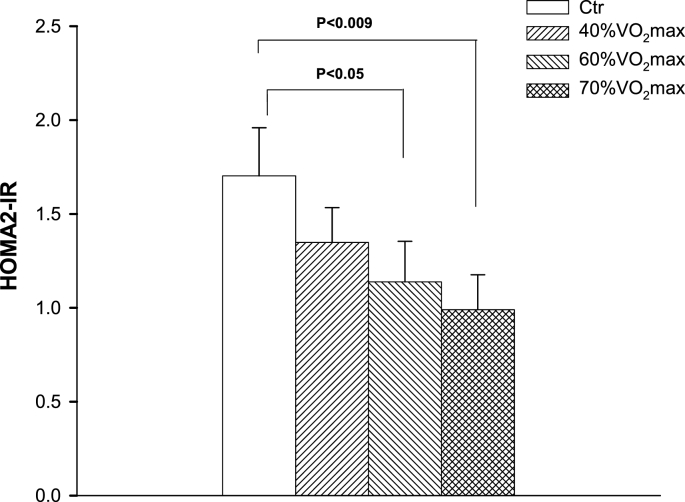
Effect of exercise intensities on HOMA2-IR (insulin resistance index). Exercise at 60 % and 70 % VO_2max_ trials significantly lowered HOMA2-IR (p < 0.005 and p < 0.01, respectively).

**Fig. 6:**
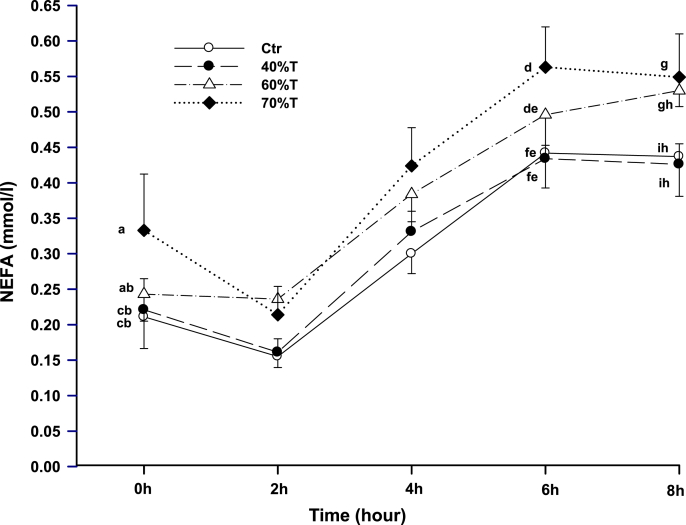
Exercise intensities and NEFA. Means not sharing a common English letter are significantly different from each other over the trials at P < 0.05. NEFA at 70 % VO_2max_ trial was significantly higher than 40 % VO_2max_ trial and the control trial at 0 h, 6 h, and 8 h after the fat meal ingestion.

The HOMA2-IR data, presented in Fig. 5, shows a significant difference in both 60 % T and 70 % T when compared to Control (p < 0.05 and p < 0.01, respectively). However, a significant difference was not observed between 40 % T and Control. Also, the exercise trials did not significantly differ from each other. Fig. 6 displays the effect of exercise intensity on NEFA concentrations of the 8 h period. At 0 h, 6 h, and 8 h, the NEFA concentrations at 70 % T were significantly (p < 0.05) higher than that in both the control and 40 % T, but there was no difference when compared to 60 % T. The other trials were not significantly different from each other.

## 4 Discussion

The main finding in the present study is that an acute bout of exercise at 40 %, 60 %, and 70 % VO_2max_ 12 h before a fat-meal intake had similar effects on attenuating PHTG. The results were indicated by TG concentration and TG area score. Secondarily, insulin sensitivity improved among all exercise trials, resulting in decreased insulin response to a fat-rich meal.

The participants selected for the study were of normal baseline characteristics with an average age of 34 years (Table 1). Fasting TG was lower than 1.69 mmol/l and the average BMI was 23.3 kg/m^2^. The high fat meal ingestion induced PHTG with TG concentration peaking at 4 h in all trials in these healthy participants. However, Ctr displayed highly exaggerated TG response than all exercise trials, which coincided with other studies that investigated moderate intensity on normolipidemic participants.^21^^,^^33^^,^^34^ Similarly, studies on individuals with metabolic conditions also exhibited higher PHTG peak in the control trial than in the exercise trials.^28^^,^^35^ High level of visceral adipose tissue also impacts the response of PHTG similar to metabolic conditions. This phenomenon was observed in overweight adolescents^19^ and adults.^36^^,^^37^ Low levels of muscle lipoprotein lipase in individuals that are obese or have a metabolic condition, such as type 2 diabetes, may influence high responses in PHTG. The activation and utilization of skeletal muscle lipoprotein lipase is speculated to attenuate PHTG response by facilitating TG and chylomicron clearance.^33^

While fasting TG is widely used for health assessments, postprandial provides a better indicator for risks of metabolic conditions.^38^^,^^39^ All exercise trials in the present study attenuated PHTG response induced by a fat-rich meal ingested 12 h later compared to the non-exercise trial. However, as seen on Fig. 3, there were no significant differences in TG concentration and TG tAUC score among the exercise trials with different intensities. Similar results were observed by Tsetsonis and Hardman^40^ that moderate intensity exercise significantly lowered PHTG. Agreement in the results were also verified by Trombold et al.^34^ and Kim et al.^21^ that exercise at intensities of 50 % and 65 % VO_2max_ mitigated PHTG response. However, Trombold et al. observed a more robust response in PHTG clearance after a high intensity exercise trial at 90 % VO_2max_.^34^ Significant TG clearance at high intensity exercises was also reported by Gabriel et al.^41^ Although the authors did not directly assess how exercise ameliorate PHTG, the possible mechanisms attributing to this difference are the reduction of hepatic VLDL-TG secretion and enhanced TG clearance due to increased skeletal muscle lipoprotein lipase activity after exercise.^35^ Additionally, exercise-induced postprandial lipemia reduction is also associated with decreased insulin levels observed in exercise trials, leading to insulin-mediated inhibition of skeletal muscle lipoprotein lipase activity, and enhanced TG clearance at this site.^42^

It is also noteworthy that intermittently accumulated exercise sessions could be equally effective in reducing PHTG compared to single continuous exercise.^17^^,^^18^ The study conducted by Kim et al. showed 33.6 % and 19.8 % PHTG reductions in moderate- (65 % VO_2max_) and low-intensity (25 % VO_2max_) intermittent exercises compared to control (no exercise), respectively, while a 17.2 % PHTG reduction was observed in moderate-compared to low-intensity exercises.^21^ In accordance, our study depicted an average of 27 % PHTG reduction (TG tAUC score) in the exercise trials than in the non-exercise trial. It is difficult to directly compare each study due to variances in participant characteristics and exercise protocols. Diets and the time of ingestions also varied among the studies. Nevertheless, there is a trend of an effective reduction of postprandial lipemia when the exercise is partaken in the moderate to high intensity range.

Exercise at low to moderate intensities demonstrated a dramatic lowering effect on insulin 2 h after meal ingestion compared with the control. At 0 h and 4 h, only 70 % T had insulin lowering effect compared to the control. This may be attributed to the highest caloric expenditure in the 70 % T as illustrated in Table 3. Higher intensity exercise utilizes more glycogen storage, which in turn induces more replenishment of the used glycogen after exercise.^43^ This may result in better plasma glucose handling, hence, lower insulin concentrations as observed in the 70 % T. Indeed, research data has revealed that exercise-induced glycogen depletion and enhanced glycogen synthase activity may contribute to the improvement of insulin sensitivity and attenuated insulin resistance.^43^, ^44^, ^45^ The results were in agreement with a similar study using hyperlipidemic participants, which demonstrated that exercising at 40–70 % intensities not only attenuated PHTG, but also lowered insulin response.^28^ Newsom et al. showed that a single session of exercise (expending 350 kcal) at a relatively low-intensity (50 % VO_2max_) was sufficient to significantly improve insulin sensitivity at least into the next day in obese adults, partially mediated by attenuated systemic fatty acid mobilization and uptake.^46^ Ryan et al. reported that 12 weeks of moderate-intensity exercise and high-intensity interval training induce similar acute improvements in peripheral insulin sensitivity and metabolic adaptations in skeletal muscles in obese adults.^32^ These findings suggest that low to moderate exercise intensities may have similar insulin lowering and sensitizing effects in various populations with metabolic disorders, such as obese and hyperlipidemic individuals.

CVD and type 2 diabetes have been associated with insulin resistance.^47^^,^^48^ Depicted by HOMA2-IR in Fig. 5, exercise intensities at both 60 % and 70 % VO_2max_ significantly attenuated insulin resistance. There was no significant attenuation from 40%VO_2max_, although a decrease in insulin resistance was observed. These results are consistent with a study by Zhang et al.,^28^ which also observed similar insulin resistance reduction after 1 h of exercise bout at 40–70 % VO_2max_. Additionally, the current study did not reveal exercise effect on fasting glucose concentration. In contrast, Zhang et al.^28^ reported that 70 % VO_2max_ exercise attenuated fasting glucose. The discrepancy may attribute to the investigated participants with hypertriglyceridemia who had abnormal average fasting glucose of 6.54 ± 0.24 mmol/l.^28^ The high fasting glucose concentration allows more of an opportunity of improvement compared to the current study.

NEFA is known to facilitate lipid oxidation by shuttling TG to be utilized. Before the fat-rich meal, NEFA concentrations were higher for 60 % T and 70 % T compared to 40 % T and the control. Because of the increased insulin immediately after the meal ingestion, NEFA concentrations fell at 2 h but steadily rose thereafter to aid in lipid oxidation. As depicted in Figs. 6 and 60 % T and 70 % T tended to demonstrate higher concentrations of NEFA at 6, and 8 h. The higher NEFA observed in 60 % T and 70 % T was attributed to higher intensity-exercise induced caloric expenditure. The enhanced caloric expenditure results in PHTG attenuation and insulin resistance reduction.^25^^,^^49^^,^^50^

The mechanism of hepatic VLDL-TG secretion and LPL-mediated clearance of TG-rich lipoproteins are greatly influenced by insulin resistance and hyperinsulinemia.^51^, ^52^, ^53^ The concentrations of PHTG and fasting TG become elevated from increased hepatic VLDL-TG secretions and inhibited LPL-mediated clearance. In general, exercise may mitigate the atherosclerosis forming events. The results of the present study suggest that exercise at low to moderate intensities for 1 h effectively diminishes PHTG (Fig. 3), insulin responses (Fig. 4), as well as insulin resistance. Muscle lipoprotein lipase may possess a role in the reduction in PHTG, with the activity having a positive association with exercise intensity.^33^ Evidence has shown that exercise can increase skeletal muscle capillarization, thus improving insulin sensitivity and glucose tolerance.^11^ The inhibition or mitigation of lipid oxidation in skeletal muscles can also be partly responsible for atherosclerosis and obesity.^54^ Exercise has been shown to be vital for the effectiveness of lipid oxidation as illustrated by our data (Fig. 6) and others.^55^

Although this study reported some innovative findings on different exercise intensities on PHTG and insulin resistance in healthy individuals, there were some limitations in this study. First, a more frequent blood sampling time (e.g. every 30 min) may provide more information on TG clearance and insulin/glucose changes responding to various exercise intensities. It would provide more information on glucose metabolism if the blood glucose time course changes were measured after the exercise trials and test meals. In addition, we did not standardize the energy intake and/or energy expenditure data in this study, and we relied on making telephone calls to remind the participants to restrain from exercising for three days prior to the trial. However, the phone calls may not completely limit the participants from exercising or similar physical activities. This might be potential confounding factors on PHTG in exercise interventions because previous study showed attenuation of PHTG was largely dependent on exercise energy expenditure.^27^ Lastly, larger sample size and more advanced statistical analysis tool (e.g. Generalized Estimating Equations) in future studies would increase the power to detect the statistical difference in repeated measured exercise trials.

In conclusion, the current study revealed that exercising at 40, 60, or 70 % VO_2max_ prior to ingesting a fat-rich meal considerably attenuates PHTG response in normolipidemic, young participants. All three intensities effectively mitigated insulin responses, notably at 2 h post-meal. The lowering effects of PHTG, insulin, and insulin resistance tended to be greater as the exercise intensity increased. The alleviating influence of exercise on PHTG and insulin response may be attributable to the energy expenditure incurred during exercise. These findings suggest that exercising at low to moderate intensity may be sufficient in preventing atherosclerosis and metabolic conditions among young, healthy, and recreationally active individuals. These results may provide exercise-oriented health promotion measures for communities with both healthy and at-risk individuals.

## Author's statement

We declare that this manuscript is original, has not been published before and is not currently being considered for publication elsewhere.

We confirm that the manuscript has been read and approved by all named authors and that there are no other persons who satisfied the criteria for authorship but are not listed. We further confirm that the order of authors listed in the manuscript has been approved by all of us.

We understand that the Corresponding Author is the sole contact for the Editorial process.

He is responsible for communicating with the other authors about progress, submissions of revisions and final approval of proofs.

## Declaration of competing interest

The author(s) have no conflicts of interest relevant to this article.
